# Efficient Grafting of Cyclodextrin to Alginate and Performance of the Hydrogel for Release of Model Drug

**DOI:** 10.1038/s41598-019-45761-4

**Published:** 2019-06-27

**Authors:** Line Aa. Omtvedt, Marianne Ø. Dalheim, Thorbjørn T. Nielsen, Kim L. Larsen, Berit L. Strand, Finn L. Aachmann

**Affiliations:** 1 Norwegian Biopolymer Laboratory (NOBIPOL), Department of Biotechnology and Food Science, NTNU - Norwegian University of Science and Technology, N-7491 Trondheim, Norway; 2 Department of Chemistry and Bioscience, Aalborg University (AAU), 9220 Aalborg, Denmark

**Keywords:** Polysaccharides, Biomedical materials

## Abstract

Controlling the rate of release of molecules from a hydrogel is of high interest for various drug delivery systems and medical devices. A strategy to alter the release profiles of soluble and poorly soluble active ingredients from hydrogels can be to combine the hydrogel forming ability of alginate with the inclusion forming ability of cyclodextrins (CyD). Here, β-CyD was grafted to alginate in a three-step synthesis using periodate oxidation, reductive amination and copper(I)-catalyzed azide-alkyne cycloaddition. A grafting degree of 4.7% mol β-CyD/mol sugar residues was obtained. The grafting degree was controlled by varying the reaction parameters where the amount of linker used in reductive amination was especially influential. Ca-alginate gel beads grafted with β-CyD showed increased uptake of the model molecule methyl orange. Release experiments showed that the grafted material had a prolonged release of methyl orange and an increased total amount of released methyl orange. These results show that the β-CyD grafted alginate is still able to form a hydrogel while the grafted cyclodextrins retain their ability to form inclusion complex with methyl orange. Further testing should be done with this system to investigate capability for drug delivery applications.

## Introduction

Hydrogels may be described as water molecules entrapped with in a hydrophilic polymer network (typically 1–3% dry matter) with the mechanical properties of a solid^1^. The hydrophilic nature of a hydrogel limits its applicability as carrier of a large range of active compounds (e.g. drugs) as they most often are poorly soluble in water. The use of hydrogels as a vehicle for the release of poorly soluble drugs is thus hampered by the hydrophilic nature of the hydrogel, which leads to limited drug loading and, consequently, limited drug release. In the case of hydrophilic drugs, high loading degrees and high release rates are largely governed by the diffusion rates of the drug. In alginate-based hydrogels, the permeability of the gel network limits the drug loading and release rate. In addition, the shape, charge and size of the molecule of interest also influence the loading degree and release rate^2^. CyDs are known to alter the apparent physiochemical properties of hydrophobic molecules and molecules with hydrophobic moieties due to the formation of guest-host complexes and can, for example, improve the bioavailability of drugs and change their release profiles from drug delivery devices^3^. In this context, CyD-grafted alginates could provide a system that combines the gelling properties of alginate with the ability of CyDs to form inclusion complexes with molecules presenting hydrophobic moieties (e.g. drugs).

Alginate is a linear polysaccharide that consists of (1 → 4) linked α-l-guluronic acid (G) and β-d-mannuronic acid (M), and can be isolated from brown algae and certain bacteria^4^. Specific sequences of monosaccharide in alginate are usually described as block structures: M-blocks consisting of consecutive sequences of M-residues, G-blocks consisting of consecutive sequences of G-residues, and MG-blocks describing sequences of alternating M and G-residues. The relative amounts of each block structure varies in different types of alginates^5^, which, together with the molecular weight, determines the different properties of alginates^2^. The single most important property of alginates is their ability to form hydrogels by crosslinking with divalent cations, such as calcium ions. While G-blocks are largely responsible for this key trait^6^, MG-blocks can also play a significant role in Ca-crosslinking^7^. Alginates are in general considered to be non-toxic and with low immunogenicity and are widely used in biomedical and pharmaceutical applications^8^. Recent studies have focused on fibrotic reactions towards alginate-based capsules where fibrosis is believed to be connected to early inflammatory responses, but the mechanisms are currently not understood^9,10^.

CyDs are macrocycles produced from starch made up of at least six α*-*(1 → 4) linked glucopyranose residues^11^. Native, unmodified CyDs with 6, 7, and 8 glucopyranose units in the macrocycle (named α-, β- and γ-CyD, respectively), are commercially available as fairly cheap bulk chemicals^12^. The CyDs can be described as truncated cones with a relatively hydrophobic cavity and a hydrophilic exterior. This gives CyDs the ability to form inclusion complexes in water with various hydrophobic molecules and moieties^11,13–16^. As drug-CyD inclusions complexes usually have a higher aqueous solubility compared with the pure drug, the apparent solubility of the drug is typically increased by CyDs. This feature, together with fast dissolution rates allows CyD to increase the bioavailability of a large range of drugs. Furthermore, by forming inclusion complexes, CyDs protect drugs from being prematurely degraded and metabolized^17^. CyDs are in general considered as biocompatible in relation to their participation as functional elements in materials and devices intended for medical uses as exemplified by recent publications^18–20^. Cyclodextrins have even shown to improve the biocompatibility of e.g. drug delivery systems^21^. CyDs have for example been used to enhance the apparent solubility of poorly water-soluble drugs in a PVP/PEG crosslinked hydrogel^22^. However, in systems of synthetic polymers such as PEG, the hydrogels are formed via covalent crosslinks between the polymer chains, often under the use of harsh chemical conditions or ultra violet light^22,23^. Since alginate crosslinks with divalent cations, the hydrogels can be formed in aqueous solution and at physiological conditions^4^.

Alginates have been used as excipients in drug delivery^24,25^, and can be chemically modified to obtain drug delivery systems with altered release rates and profiles^25^. Grafting alginate with CyDs should enable the hydrogel to host higher concentrations of poorly soluble drugs and other active small molecules that otherwise would readily diffuse out of the gel. Furthermore, molecules capable of inclusion complex formation with CyDs should display sustained release properties (irrespective of their solubility) compared to non-modified alginate gels.

Various strategies have been used to covalently attach β-CyD to polymer chains, including photo initiated free radical polymerization for synthetic polymers^26^ and carbodiimide chemistry, reductive amination, click chemistry or combinations of these for polysaccharides^27–31^. In the case of alginates, carbodiimide chemistry has been used to link CyD to its carboxyl group either via an amine functionalized CyD^28,29,32,33^ or via a linker having a primary amine^27^. Carbodiimide chemistry is commonly used to conjugate various primary amines to the carboxylate groups in alginate^34^. However, the reaction gives by-products that have been shown to associate with the alginate^35^. Also, the grafting degree of alginate using carbodiimide chemistry has been limited to 0.1 to 0.2% of monomers substituted for peptides^8,35^. The secondary hydroxyl-groups on alginate have also been used to covalently graft α-CyDs on the polysaccharide backbone, by using the cyanogen bromide method^36^. In our previous work, periodate oxidation and reductive amination was shown to be an efficient alternative to carbodiimide chemistry for grafting of peptides^37^. This approach resulted in high and tunable grafting degrees without by-product formation. Hence, grafting up to the level of degree of oxidation (e.g. 8% grafting for 8% oxidized material) has been obtained^37^. The copper(I)-catalyzed azide-alkyne cycloaddition (CuAAC) reaction has been used to covalently bind β-CyD to an alkyne-linked dextran^31^. The CuAAC-reaction is a versatile click-reaction that gives 1,4-disubstituted-1,2,3-triazoles as the end product^38–40^.

Despite recent developments of various methods, grafting of suitable functional moieties to alginate in aqueous solution is still far from optimal. Development of a controlled and tunable grafting method without by-product formation in aqueous solution would be highly advantageous. The aim of the present paper is to combine the latest developments in polysaccharide-grafting methods with aqueous-solution click-chemistry to graft CyDs to alginates^31,37^.

## Materials and Methods

### Materials

Alginates from *Laminaria hyperborea* stipe (F_G_ = 0.7, N_G>1_ = 11–14, ${\bar{{\boldsymbol{M}}}}_{{\boldsymbol{w}}}$ = ~100 kDa) were obtained from FMC Health and Nutrition, Sandvika, Norway. 6-O-monodeoxy-6-monoazido-β-CyD (N_3_-β-CyD) was synthesized as described previously^31^. Tris(benzyltriazolylmethyl)amine (TBTA) was synthesized according to literature^41^ (used for sample B and C) or bought from Sigma Aldrich (used for sample A). All other chemicals were obtained from commercial sources and were of analytical grade. Deionized water (water purified with the MilliQ system from Millipore, Bedford, MA, USA) was used in all solutions.

### Coupling of linker to alginate

**Figure 1:**
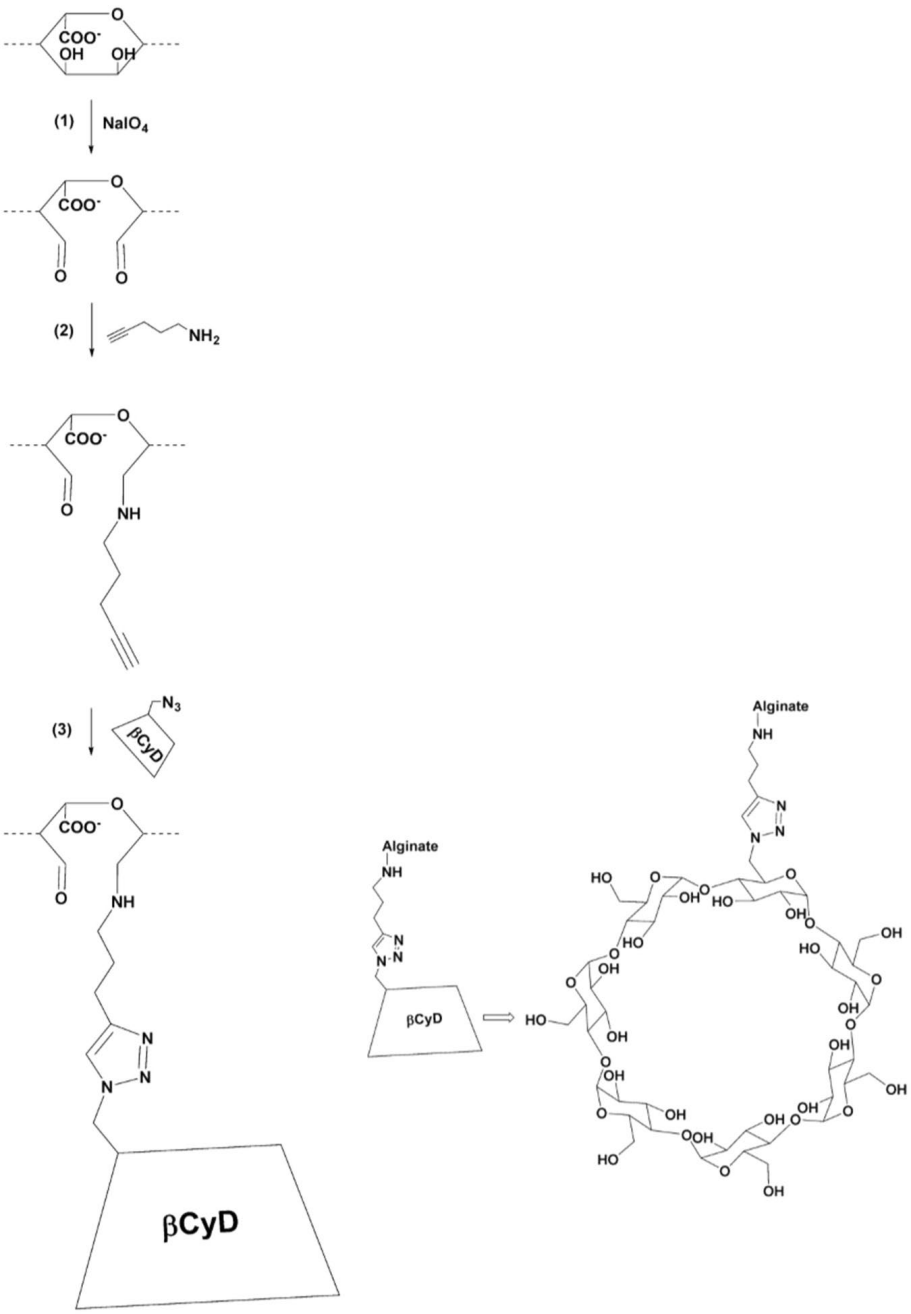
Scheme of the three-step synthesis of alginate (here shown for a G residue) grafted with β-CyD; (1) partial oxidation of alginate with metaperiodate, (2) grafting of the alkyne linker to either C2 or C3 of the oxidized alginate residues^37^ (3) grafting of N_3_-β-CyD to the alkyne linker with the Cu(I)-catalyzed azide alkyne cycloaddition click-reaction^31^. β-CyD is not appropriately scaled compared to the alginate.

Periodate oxidation followed by reductive amination was used to covalently bind the linker 4-pentyn-1-amine to the alginate chain (see Fig. 1, step (1) and step (2)). In step (1) the alginate was partially oxidized using periodate ions (IO_4_^−^), based on previously published protocols^42–44^.

Alginate was dried overnight in a desiccator. The weight of the dried alginate was adjusted for water content (10% residual water)^45^ and dissolved in water corresponding to a final concentration of 7.0–8.8 mg/ml, depending on the viscosity of the solution. n-propanol was added to the samples to a final concentration of 10% (v/v), followed by degassing with nitrogen gas (N_2_). Henceforth, the samples were protected from light. Sodium (meta)periodate (NaIO_4_) was added from a freshly made stock solution of 0.25 M to a periodate/monomer molar ratio (P_0_) = 0.08. The samples were incubated at 4 °C under gentle mixing until the reactions had run to completion (46–72 hours). To confirm that the reactions had run to completion (all IO_4_ consumed), a titration test was performed: 0.5 ml of the reaction mixture was mixed with 5 ml 0.5 M cold phosphate buffer (NaH_2_PO_4_), pH 7.0 and 0.75 ml 60% (w/v) potassium iodide (KI). If unreacted periodate was present, this would result in yellow coloration (no color change would be observed if all periodate has been consumed). The solutions were then titrated with 2.5 mM sodium thiosulfate (Na_2_S_2_O_3_). Close to the titration end-point, a few droplets of 10 mg/ml starch was added to the samples giving a blue color. Titration was then continued until the blue color disappeared.

Finally, the samples were dialyzed against deionized water until the measured conductivity was below 4 μS. All oxidized alginates were lyophilized after dialysis and thereafter stored at −18 °C.

In step (2) (Fig. 1) the linker, 4-pentyn-1-amine was covalently linked to the oxidized alginate by reductive amination based on the protocol developed by Dalheim *et al*.^24^. Periodate oxidized alginate (POA) was dissolved in deionized water and methanol was added according to final concentrations of 3 mg/ml and 12% (v/v) respectively. 1.2 M 4-pentyn-1-amine in MeOH was added to a concentration of 6 mM or 24 mM, corresponding to 5 and 20 molar equivalents, respectively, of the linker relative to the amount of oxidized residues (P_0_ = 0.08). 0.25 M 2-picoline borane complex in MeOH was then added according to a final concentration of 24 mM (20 molar equivalents relative to P_0_). The pH was adjusted to 5.8 with 1 M acetate buffer, pH 5.0. The reactions were incubated at room temperature under gentle mixing for 48–96 hours. The samples were dialyzed (MWCO 12–14 000 kDa) against two shifts of seven liters 50 mM NaCl and subsequently against deionized -water until the conductivity was below 4 μS. The final products were freeze dried and stored at −18 °C until further use.

### Grafting of β-CyD to alginate

The grafting of β-CyD to alginate was achieved by using the Cu(I)-catalyzed azide-alkyne cycloaddition click-reaction^31^, see Fig. 1, step (3). Alginate with covalently bound 4-pentyn-1-amine (product step (2)) was dissolved in deionized water to 3.5–7.4 mg/ml. In the following procedure, the amount of added reactants is given as molar equivalents relative to the theoretical oxidation degree 8%. Two equivalents of N_3_-β-CyD were added into the alginate solution. Dimethyl sulfoxide (DMSO) was then added slowly to a final concentration of 40% (v/v). Freshly made 0.1 M sodium ascorbate was added to a final concentration of 0.3 equivalents and thereafter 0.11 equivalents of 0.02 M TBTA in DMSO were added. The mixture was deoxygenated with nitrogen gas for about five minutes, and subsequently 0.1 equivalents of 0.06 M CuSO_4_ were added. Thereafter, the mixture was again deoxygenated for about three minutes. The sample vials were closed, and the reaction mixture incubated at 50 °C under gentle mixing for 40–51 hours. All samples were dialyzed first against 50 mM NaCl and then against deionized water, as described above, until conductivity was below 2 μS and freeze dried.

To remove residual copper ions, the β-CyD grafted alginate was treated with Ambersep GT74 resin. 2–15 mg/ml β-CyD grafted alginate in deionized water was added to 8 g resin per g polymer. Samples were shaken for 24–72 hours, and the resin was removed by filtration. Samples were thereafter dialyzed, lyophilized and stored at −18 °C.

### NMR

All alginate samples were subjected to a mild acid hydrolysis as previously described^46^ to reduce the viscosity prior to NMR analysis. Approximately 10 mg sample was dissolved in ~600 μl 99.9 atom % D_2_O together with 5 μl 3-(trimethylsilyl)-propionic-2,2,3,3-*d*4 acid sodium salt (TSP; chemical shift reference) and 20 μl 0.3 M triethylene-tetra-amine hexaacetate (TTHA; chelate divalent cations). The NMR experiments were carried out on a BRUKER AVIII-HD 600 spectrometer equipped with TXI (H/C/N) probe or a BRUKER Avance DPX 400 spectrometer (Bruker, BioSpin AG, Fällanden, Switzerland) equipped with a 5 mm z-gradient DUL (C/H) probe). The amount of 4-pentyn-1-amine linker and β-CyD grafted to the alginate was determined from 1D ^1^H spectra, recorded at 90 °C on the 400 MHz spectrometer for all samples.

Diffusion-Ordered SpectroscopY (DOSY) was used to measure the diffusion of the coupled products. A 2D DOSY was measured using a Bruker BioSpin stimulated echo pulse sequence with bipolar gradients (STEBPGP). Gradient pulses of 2 ms duration (δ) and 32 different strengths varying linearly from 0.03 to 0.57 T·m^−1^ were applied and the diffusion delay (Δ) was set to 80 ms. The DOSY spectrum was recorded at 25 °C on the 600 MHz spectrometer. The spectra were recorded using the TopSpin software versions 1.3, 2.1 or 3.2 (Bruker BioSpin AG, Fällanden, Switzerland) and processed and analyzed with the TopSpin software versions 3.0 and 3.2 (Bruker BioSpin AG, Fällanden, Switzerland).

### SEC-MALS

The molecular weight of the grafted alginates was measured with Size Exclusion Chromatography with MultiAngle Light Scattering detection (SEC-MALS) according to previous published protocols^43^. The setup consisted of a mobile phase reservoir, an on-line degasser (Degasi Classic, Biotech), an HPLA isocratic pump (LC-10AD, Shimadzu), an autoinjector (SCL-10A VP, Shimadzu), a precolumn and one or two serially connected columns (TSK 6000 PW and TSK gel PWH guard column, Toso Haas, for the stipe alginate and TSK 4000 + 2500 PWXL for the modified alginates). The column outlet was connected to two serially connected detectors, a multiangle laser light scattering photometer (λ_0_ = 0.633 nm) (Dawn DSP, Wyatt, USA) followed by a differential refractometer (P-10 cell) (Optilab DSP, Wyatt, USA). 0.15 M NaNO_3_ with 0.01 M EDTA, pH 6.0 was used as the mobile phase for the stipe and partially oxidized alginate. 20% acetonitrile was added to the mobile phase for the alginate grafted with linker and β-CyD. The samples were dissolved in the mobile phase and filtered (pore size 0.8 μm) prior to injection. The analysis was carried out at ambient temperature with a flow rate of 0.5 ml/min. Injection volume and sample concentration was adjusted to obtain an optimal light scattering signal without influencing the RI profile (overloading). Astra software v. 6.1.1 (Wyatt, USA) was used to collect and process the obtained data, using a refractive index increment (dn/dc_µ_) of 0.150 ml/g for alginate samples^43^.

### Hydrogel formation of β-CyD grafted alginate and release of methyl orange

Hydrogel formation of the β-CyD grafted alginate was performed by dripping the alginate solution into a CaCl_2_ solution (100 mM). For the release experiment, four different samples of 1.8% (w/v) alginate solutions were made; one with only unmodified *L*. *hyperborea* stipe alginate, one where the unmodified alginate was mixed with free β-CyD (1.7 mg/ml), and two where unmodified alginate was mixed with 25% and 50% (w/w) β-CyD-grafted alginate, respectively). The pH of the alginate solutions was adjusted to 5.5–6.0. The weight of the β-CyD was taken into account in making the 1.8% (w/v) solutions with grafted alginates. Beads were made by dripping 3 ml of the alginate solution with a pipette into 50 ml of 100 mM CaCl_2_ with 0.15 mM methyl orange, pH 3.5. The beads were left in this solution for 13 hours to ensure saturation of both gelling Ca^2+^ ions and methyl orange.

The release of methyl orange from the beads was evaluated through a series of saline treatments, as follows. The gel beads were immersed in 18 ml 0.9% (w/v) NaCl and left on a turn-over table for one hour (first saline treatment). The beads were then recovered from the solution and immersed in another 18 ml 0.9% (w/v) NaCl (second saline treatment). This procedure was performed for a total of six times. The concentration of methyl orange in the saline treatment solutions (after removal of the beads and adjusting the pH to 8–10) was measured as absorbance at 460 nm in a Nuncleon Flat Bottom Black Polystyrol 96-well plate (Thermo Fischer Scientific) using a multifunctional plate reader (Infinite 200 PRO, TECAN). Absorbance was converted to concentration using a calibration curve for absorbance at 460 nm as a function of the concentration of methyl orange in 0.9% (w/v) NaCl. Methyl orange can be used as an indicator due to its color change at different pH values: At acidic pH methyl orange gives a red solution, while at alkaline pH the compound gives a yellow color (the pK_a_ value for methyl orange is 3.49^47^. The solutions for the calibration curve were also pH-adjusted so that the predominant form of methyl orange was the same as for the saline treatment solutions.

## Results and Discussion

β-CyD was grafted to alginate (F_G_ = 0.7) using a three-step strategy as shown in Fig. 1. The initial, (1) partial periodate oxidation, was followed by (2) grafting of a linker (4-pentyn-1-amine) by reductive amination and (3) grafting of β-CyD to the linker by the Cu(I)-catalyzed azide-alkyne cycloaddition click-reaction.

### Tunable grafting of linker to alginate

The degree of oxidation can be used to tune the final degree of grafting. In step (1) of the synthesis (Fig. 1), the alginate was subjected to an amount of periodate corresponding to a degree of oxidation of 8% (P_0_ = 0.08), which was a compromise between preserving the hydrogel formation and sufficient grafting. Higher degrees of oxidation can be obtained by adding more periodate at the expense of gelling properties^48,49^. The completeness of the reaction was checked by titration, showing that no unreacted periodate was present in the reaction mixtures. The partially oxidized alginate was also analyzed by ^1^H NMR-spectra of *L*. *hyberborea* (Supplementary Information A).

The linker chosen here for grafting CyD to alginate was based on previous work done on synthesizing β-CyD-dextran polymers^31^ and the grafting of amine-moieties to partially oxidized alginate^37^. In addition to using a linker to graft CyDs to alginate, an attempt to shorten the time of synthesis was also performed with partially oxidized alginate directly reacting with NH_2_-CyDs using reductive amination. However, grafting NH_2_-βCyDs or triazole-propylamine-βCyDs directly to partially oxidized alginate was not successful. Therefore, the linker 4-pentyn-1-amine was chosen as it had the required amine-group for use in the reductive amination reaction as well as an alkyne group which was essential for the click-reaction. Furthermore, it was hypothesized that 4-pentyn-1-amine would have long enough linker to offer easy access to the alkyne moiety during the click reaction, as well as giving sufficient accessibility to the CyD cavity after grafting without affecting the gelling properties of the alginates significantly.

**Figure 2:**
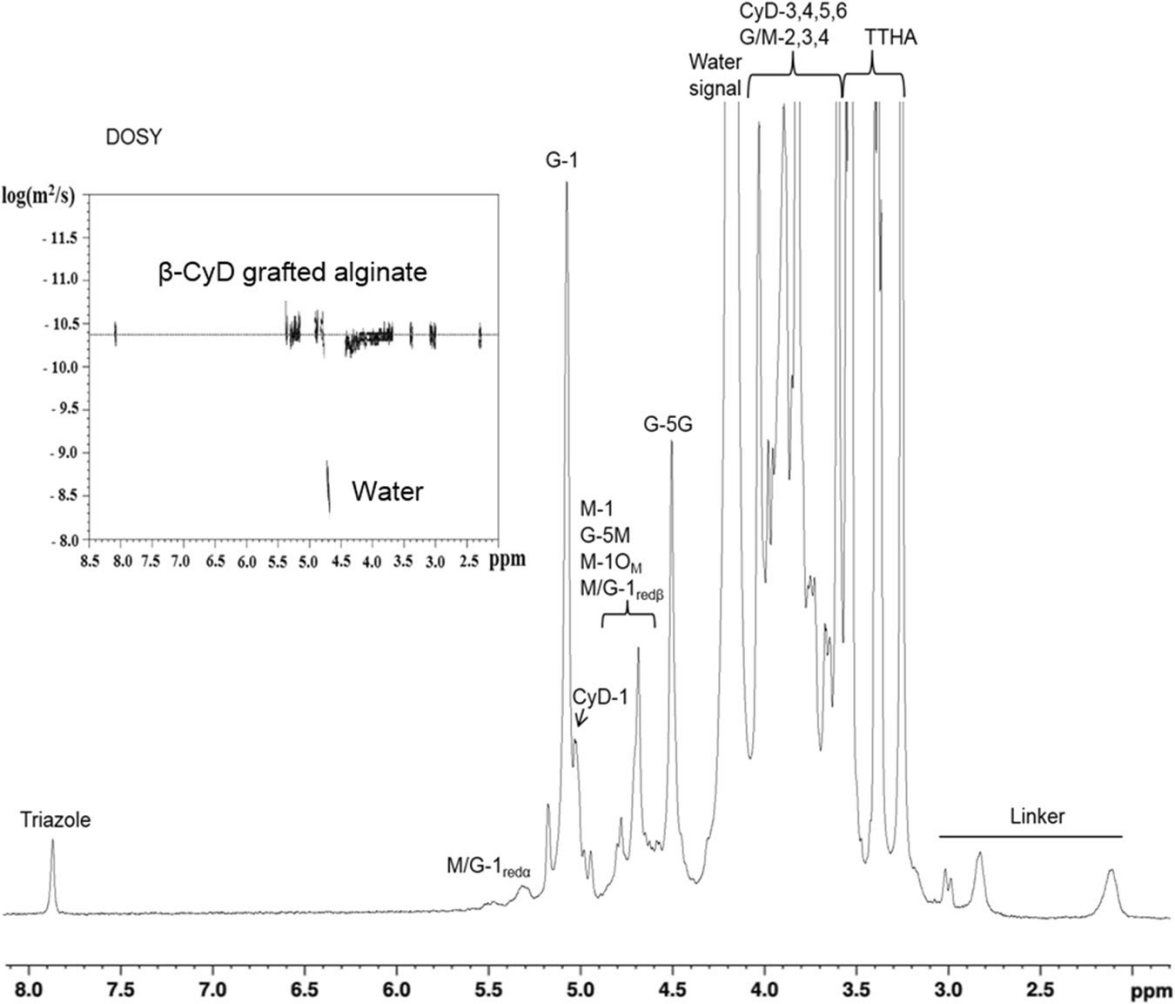
^1^H NMR and DOSY (upper left corner) of partially oxidized alginate grafted with β-CyD. The assignment of the protons of the different chemical groups are indicated. The ^1^H NMR-spectrum was recorded at 400 MHz and 90 °C in D_2_O. The DOSY spectrum was recorded at 600 MHz and 25 °C in D_2_O. Dotted lines indicates the diffusion coefficient of the molecules.

The degree of grafting for the linker in step (2) was analyzed by NMR spectroscopy and calculated as % mol linker/mol uronic acid residues, by comparing the integrals of the anomeric (H-1) protons of alginate and one of the peaks originating from the linker (see Fig. 2). An overview of the results for the reductive amination is shown in Table 1.

**Table 1:** Degree of grafting for the reductive amination and click-chemistry reactions with varying reaction parameters.

	Sample	A	B	C
Reductive amination	Linker (molar equivalents relative to P_0_)	5	20	20
	W_(POA)_ (g)	3.0	0.14	0.10
	Reaction time (hours)	96	48	84
	Substitution (% mol linker/mol monomer)	1.6	2.2	5.6
	Yield (g)	2.9	0.14	0.11
Click-reaction	Reaction time (hours)	51	48	40
	W_(POA-linker)_ (g)	2.0	0.14	0.070
	Concentration of alginate (mg/ml)	7.2	7.4	3.5
	Substitution (% mol CyD/mol linker)	100	90	84
	Yield (g)	2.5	0.097	0.067
	Final grafting degree (% mol CyD/mol uronic acid residues)	1.6	2.0	4.7

The influence of the amount of linker and reaction time was investigated for the reductive amination reaction and concentration of alginate and reaction times for the click-reaction. The final degree of CyD grafted to the partially oxidized alginate is given.

Parameter selection for the reductive amination reaction was based on a recent study where RGD-peptides were grafted to periodate oxidized alginates using reductive amination^37^. The reductive amination reaction is pH dependent and the optimal pH is, in many cases, a compromise to achieve efficient protonation of the carbonyl group and simultaneous deprotonation of the amino group^50^. In our recent work, the optimal pH for coupling of amino acids and peptides to periodate oxidized alginates was found to be 5.8^37^. However, the pK_a_ values of the terminal amino group in peptides is in the range 6.8–8.0 while the pK_a_ value of the amino group on many aliphatic primary amines is similar to the pK_a_ of 4-pentyn-1-amine,10.6^51^, thus affecting the optimal pH of the reaction. Nevertheless, pH 5.8 was also used in this study as it has been observed that protonation of the carbonyl oxygen is more important to the reductive amination reaction than deprotonation of the amino group^37^. To compensate for the suboptimal pH, a higher molar equivalent of the linker relative to the concentration of oxidized residues, as well as a prolonged reaction time, were used. Increasing the concentration of 4-pentyn-1-amine to 20 molar equivalents resulted in a grafting degree of 5.6%. This demonstrates that the amount of alkyne linker grafted to the alginate chains can be tuned by varying the concentration of the substituent. Increasing the reaction time from 48 to 84 hours (using 20 molar equivalents of substituent) also resulted in a higher degree of grafting.

### Efficient click-chemistry for the grafting of β-CyD to alginate

Figure 2 shows a typical ^1^H NMR-spectra of β-CyD grafted alginate (product of step (3)). The linker, (4-pentyn-1-amine) gives rise to signals in the region 2.0–3.0 ppm and the click reaction between 4-pentyn-1-amine and N_3_-β-CyD results in a triazole unit (see Fig. 1, step (3)) which gives a distinguishable singlet peak at 7.8 ppm. The presence of this peak clearly indicates that the click reaction was successful. Furthermore, the β-CyD grafted alginate was analyzed by DOSY to indirectly verify that β-CyD was covalently linked to the partially oxidized alginate^37^. As can be seen in Fig. 2 (insert), signals from both the linker and the triazole group appear to have the same diffusion coefficient as the alginate, indicating that they are covalently coupled. DOSY also confirms that non-reacted components were removed.

The amount of β-CyD bound to the linker was calculated as % mol β-CyD/mol linker based on the ^1^H NMR-spectra recorded for the samples after complete synthesis, using the integral of one peak for the linker and the integral of the triazole singlet peak. The concentration of alginate and reaction time were varied to study the effect on the click-reaction (Table 1). Increasing the reaction time from 40 to 48 hours and the concentration of 4-pentyn-1-amine linked alginate from 3.5 to 7.4 mg/ml increased the grafting degree from 84% (sample C) to 90% (sample B). Further increasing the reaction time in step (3) from 48 hours to 51 hours, using a concentration of 7.2 mg/ml alginate (sample A), gave full substitution of CyD on the linker (100% (mol CyD/mol linker). Taking into account the starting degree of grafting of the linker (see Table 1), this corresponded to a final degree of grafting (% mol β-CyD/mol uronic acid residues) of 1.6% for sample A, 2.0% for sample B, and of 4.7% for sample C. These results show that the copper-catalyzed click-reaction is very efficient and robust, which is in line with observations reported in literature^40^. Consequently, the most important factor for determining the final degree of grafting is the amount of alkyne linker grafted to the alginate chain.

### Molecular weight

The weight- and number-average molar mass for the unmodified stipe alginate, periodate oxidized stipe alginate (POA), linker grafted POA (POA-linker; product of step (2)) and βCyD grafted POA (POA-βCyD; product of step (3)) are presented in Table 2. The concentration profiles and molecular weights are given in Supplementary Information B.

**Table 2:** Molecular weight data derived from SEC-MALS data: weight- and number-average molar mass (${\bar{{\rm{M}}}}_{{\rm{w}}}$ and ${\bar{{\rm{M}}}}_{{\rm{n}}}$).

Sample	${\bar{{\bf{M}}}}_{{\bf{w}}}$ (kDa)	${\bar{{\bf{M}}}}_{{\bf{n}}}$ (kDa)
*L*. *hyperborea* Alginate	135	79
POA	89	48
POA-linker	63	36
POA-βCyD	70	42

POA: partially oxidized alginate. POA-linker: POA coupled with 4-pentyn-1-amine. POA-βCyD: β-CyD-grafted alginate.

As seen in Table 2 both the oxidation and the reductive amination reaction was accompanied with some degradation of the alginate. Similar results have also been observed^37^ previously. Partial periodate oxidation gives the alginate polymer a more flexible nature due to the ring opening of the oxidized residues^42,43,52^. The ring opening further enhances the susceptibility to degradation of the alginate^53^. However, after the reduction has taken place, the stability of the polymer at physiological pH is expected to be comparable to that of native alginate^54^. Degradation caused by the click-reaction is not apparent from the weight-average molar mass as seen in Table 2. Indeed, an increase in molar mass is observed for the β-CyD-grafted alginate. If no degradation occurs during the click-reaction, an increase in molar mass is expected as a result of the grafting of CyDs on the alginate.

### Hydrogel formation of β-CyD grafted alginate and release of methyl orange

The objective of this study was to combine the gelation ability of the alginate with the capability of CyDs to form inclusion complexes. Therefore, a proof-of-concept study was carried out to evaluate whether the new grafted material retained the intrinsic properties of both the alginate and the CyD. The β-CyD-grafted alginate was by itself able to form Ca-alginate beads. However, the gel was very labile and easily deformed upon handling. The disruption of the alginates’ gelling ability can largely be ascribed to the structural changes in the alginate molecule upon periodate oxidation, namely opening of the sugar ring between C2 and C3, which forms flexible “hinges” in the polymer chain^42,43,52^. This basically disrupts the G-blocks sequences and, consequently, the polymers’ potential for ionic crosslinking. Furthermore, the oxidation of G-units have previously been found to occur faster than the oxidation of M-units in the alginate chain^55^, underlining that the effective length of the ion-binding G-blocks is most likely shortened due to the oxidation. Therefore, Ca-alginate beads in this study were made from a mixture of the β-CyD-grafted alginate and a strong gelling alginate (unmodified *L*. *hyperborea* stipe alginate). This resulted in gel beads with good initial integrity suitable for release studies.

**Figure 3:**
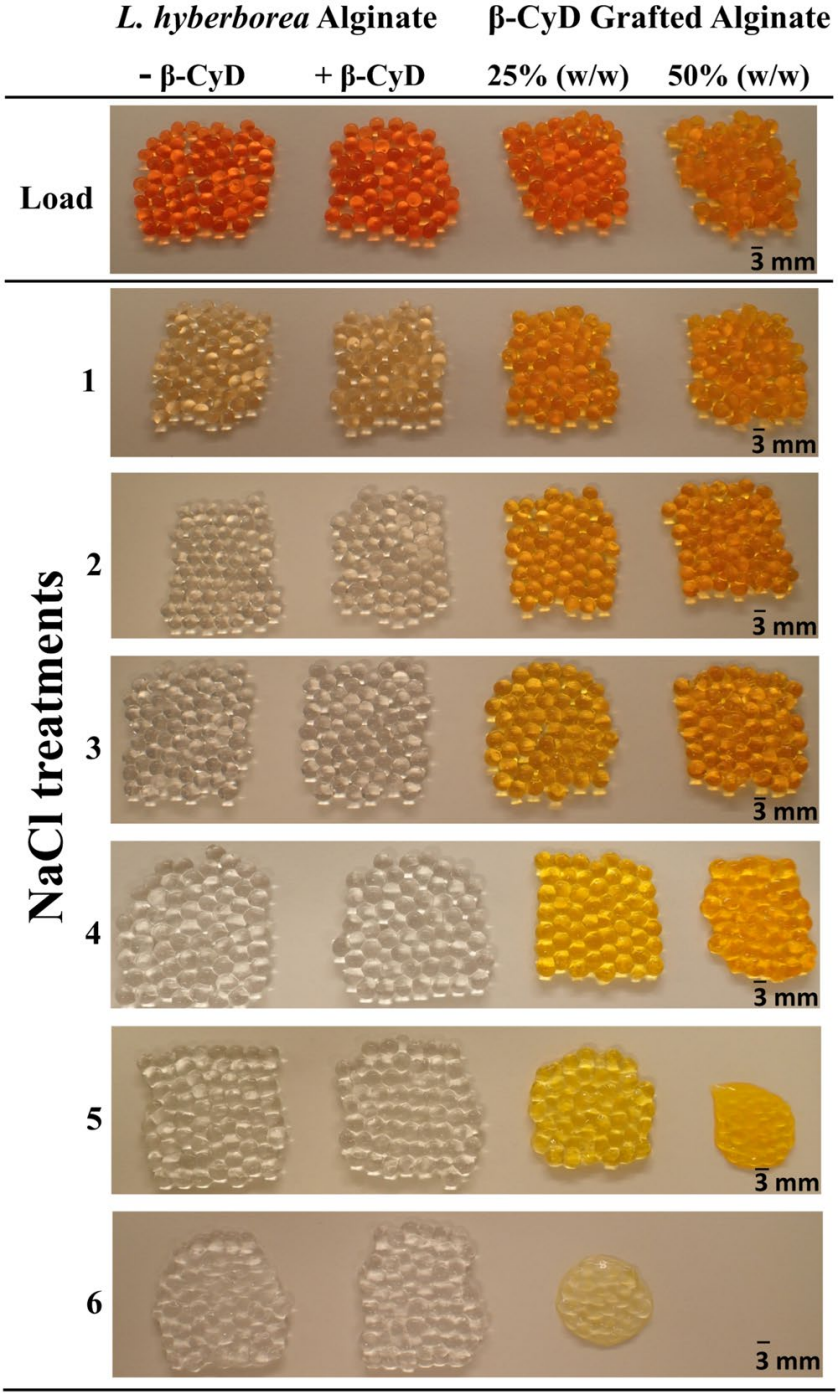
Ca-alginate beads made of unmodified *L*. *hyberborea* stipe alginate (−/+free β-CyD), and beads containing β-CyD grafted alginate mixed with unmodified *L*. *hyberborea* stipe alginate in two different ratios. The pictures were taken after removal of all the beads from the load-solution containing methyl orange (load) and gelling ions, and after immersion in consecutive solutions of 0.9% (w/v) NaCl (treatment 1–6).

The stability of the gel beads was evaluated by visual inspection of the beads after immersing them in consecutive saline solutions. The beads containing β-CyD-grafted alginate were more labile and dissolved faster compared to pure alginate beads (Fig. 3). Disintegration was visible for beads with 50% (w/w) β-CyD-grafted alginate after the fourth saline treatment, while beads made of 25% (w/w) were more stable. In contrast, the beads made from only unmodified alginate with or without free β-CyD remained largely intact after all six saline treatments. This clearly shows that the content of grafted material influences the stability of the beads. Swelling and disruption of the Ca-alginate gel beads when placed in saline solutions was expected, as calcium-ions are exchanged by sodium-ions, leading to a weakening of the gel network followed by swelling^56^. Both reduction of G-block sequence length and numbers upon grafting and reduced molecular weight of the polymer can reduce gel strength of the grafted material^2^, resulting in weaker and less stable gels compared to the unmodified alginate. The bulky nature of the CyD may also destabilize the gel network by preventing the formation of junction zones due to steric hindrance^27^. Swelling of alginate drug delivery systems have been shown to depend on which body fluid they come into contact with^24^. A destabilized gel may be favorable in a system where clearance of the gel over time is wanted. However, in order to stabilize by the alginate gel, other gelling ions (e.g. Ba^2+^ or Sr^2+^) could be used, which are known to form stronger crosslinks and depend on shorter G-blocks^57,58^. In addition, using a chemo-enzymatic strategy and introducing the G-blocks after the chemical modification may lead to a more stable gel^35,59^. Degradation of the alginate or the grafted alginate is expected to be negligible since alginate hydrolysis is slow at neutral pH and so is the hydrolysis of grafted alginates^54^.

The ability of β-CyD-grafted alginates to form inclusion complexes was studied using methyl orange as a model compound. β-CyDs can form inclusion complexes with methyl orange where the inclusion complex in neutral/basic media can be both 1:1 (K_a_ = ~3000 M^−1^) and 1:2 (K_a_ < 100 M^−1^)^60^. The association constant for the 1:2 inclusion complex is low compared to the 1:1 inclusion complex and can therefore be neglected. Visual inspection of the beads after each saline treatment revealed that the beads containing the β-CyD-grafted alginate retained their color longer than both reference beads (unmodified alginate with and without free β-CyD, Fig. 3). After the third saline treatment, there was no observable color left in the reference beads, while the β-CyD-grafted alginate beads retained their color until dissolution of the beads. This strongly suggests β-CyD retain the ability to form inclusion complexes with methyl orange is upon grafting to alginates.

**Figure 4:**
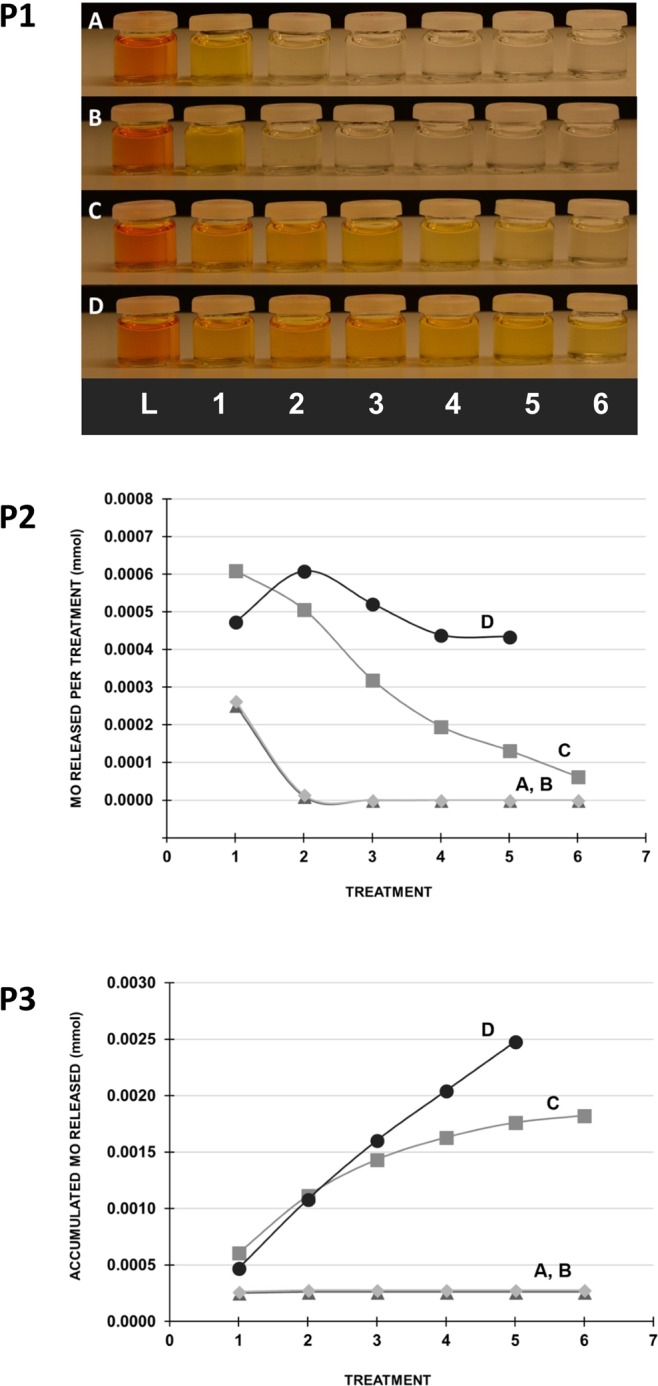
Release of methyl orange (MO) from Ca-alginate beads in consecutive saline treatments (0.9% (w/v) NaCl, one hour). Upper panel (P1): Picture of loading solutions (L) and saline treatment solutions (numbers 1–6) after removal of the beads. Middle panel (P2): Quantified amount of MO in the treatment solutions. Lower panel (P3): Accumulated release of MO. Beads made of unmodified alginate (A), unmodified alginate + free β-CyD (B), 75% (w/w) unmodified alginate +25% (w/w) β-CyD-grafted alginate (C) and 50% (w/w) unmodified alginate +50% (w/w) β-CyD-grafted alginate (D). Guidelines are drawn between the measurements.

The release of methyl orange from the beads was assessed by the content in the saline solutions (Fig. 4). Visual inspection clearly showed a continued discoloration from the beads with β-CyD-grafted alginate. In contrast to this, saline solutions from beads not containing β-CyD or free β-CyD were near to colorless after the first saline treatment. The concentration of methyl orange in the saline solutions was quantified by measuring the absorbance at 460 nm. The quantitative measurements confirmed the observations that the release of methyl orange continued throughout the saline treatments for the beads with β-CyD-grafted alginate. More methyl orange was released from beads with 50% β-CyD-grafted alginate than beads with 25% β-CyD-grafted alginate. However, the beads with 50% β-CyD-grafted alginate were largely destabilized by extensive saline treatment and were fully dissolved in the last treatment solution, as discussed previously. Hence, it cannot be excluded that some of the methyl orange in the treatment solution was still bound to the β-CyD-grafted alginate that dissolved from the gel bead.

Earlier studies have shown that the inclusion complex between β-CyD and methyl orange has a different absorbance than pure methyl orange^60^, which could influence the results when quantifying the methyl orange in the saline solutions. However, in this study no significant changes in absorbance were observed. The association constants of polymer-bound CyD towards various guest molecules has previously been shown to change compared to free CyD and guest molecule alone^31,61^. This has not yet been tested with the β-CyD-grafted alginates.

The data indicate that the beads containing β-CyD-grafted alginate were able to absorb significantly more methyl orange compared to the beads made of only alginate and alginate with free β-CyD. Assuming a homogeneous distribution of methyl orange in the loading solution containing the alginate beads (140 µmol/L), the amount of methyl orange in the gel volume (3 mL) would be 0.4 µmol. As seen in Fig. 4, the released methyl orange from beads with 50% β-CyD-grafted and 25% β-CyD-grafted alginate is 6.3 and 4.5 times higher, respectively, indicating an accumulation of methyl orange in the beads with β-CyD-grafted alginate proportional to the amount of grafted material.

The ratio of methyl orange released from the beads and the amount of grafted β-CyD in the modified alginate beads can give further information on the release system. The amount of grafted β-CyD was 1.0 and 2.0 µmol for the beads with 25% and 50% β-CyD-grafted alginate, respectively. The total amount of methyl orange released from the 25% modified alginate beads was 1.8 µmol. This gives a ratio between the cyclodextrin and methyl orange of approximately 1:2. In the beads where 50% of the alginate was modified, 2.5 µmol is released and hence closer to 1:1 ratio between β-CyD and methyl orange.

The gel beads with unmodified alginate and free β-CyD displayed a similar release profile of methyl orange as those made of unmodified alginate only (Fig. 4), indicating that the free β-CyD did not retain methyl orange in the gel network as observed for the grafted β-CyD. NMR analysis of the methyl orange load solution for the beads made from alginate and free β-CyD showed that β-CyD (1135 Da) had leaked out of the beads and into the surrounding solution (data not shown). Previously, IgG (150 kDa) has been shown to diffuse into Ca-alginate microbeads^62^. Hence, the alginate gel by itself has limited ability to immobilize smaller molecules as also demonstrated in this study. However, via grafting of β-CyD, methyl orange could be retained in the beads before being released upon treatments with saline solution. This system could be interesting for different fields and further studies are needed to understand its full potential, such as drug delivery, tissue engineering and water treatment with alginate binding divalent ions and CyD binding smaller non-polar molecules^63^.

## Conclusion

Alginate was successfully grafted with β-CyD through a three-step synthesis, combining the gelation ability of the alginate with the inclusion complex ability of the cyclodextrins. The obtained degree of grafting ranged from 1.6–4.7% mol β-CyD/mol uronic acid residues, depending on the applied reaction parameters. The chosen synthesis methodology allows the degree of grafting to be regulated, resulting in a material that has tunable properties. The final degree of grafting was shown to depend largely on the amount of linker (4-pentyn-1-amine) grafted to periodate oxidized alginate. A reduction in molecular weight was observed during the oxidation and reductive amination step. The proof-of-concept study showed that the grafted CyDs retained their ability to form inclusion complex with methyl orange. Gel beads made from a mixture of β-CyD-grafted alginate and unmodified alginate had an increased and prolonged release of methyl orange compared to non-modified beads. The stability of the Ca-alginate gel beads was also affected by varying the amounts of the grafted and unmodified alginates. Altogether, the β-CyD grafted alginate can potentially be used as an adaptable release system and further investigating is being done on the use of β-CyD-grafted alginate for controlled drug release.

## Supplementary information

Supplementary info
