# Supplementary material for: Efficient Grafting of Cyclodextrin to Alginate and Performance of the Hydrogel for Release of Model Drug
Source: Sci Rep. 2019 Jun 27;9:9325. doi: 10.1038/s41598-019-45761-4 (PMC6597533; doi:10.1038/s41598-019-45761-4)
Supplement: Supplementary file 1 — Supplementary info [file 41598_2019_45761_MOESM1_ESM.docx]

**Efficient Grafting of Cyclodextrin to Alginate and Performance of the Hydrogel for Release of Model Drug.**

*Line Aa. Omtvedt^1,#^, Marianne Ø. Dalheim^1#^, Thorbjørn T. Nielsen^2^, Kim L. Larsen^2^, Berit L. Strand^1^, and Finn L. Aachmann^1*^*

^1^Norwegian Biopolymer Laboratory (NOBIPOL), Department of Biotechnology and Food Science, NTNU - Norwegian University of Science and Technology, N-7491 Trondheim, Norway.

^2^Department of Chemistry and Bioscience, Aalborg University (AAU), 9220 Aalborg, Denmark.

^#^Shared authorship

*Corresponding author Finn L. Aachmann – [finn.l.aachmann@ntnu.no](mailto:finn.l.aachmann@ntnu.no)

Supplementary Information A – ^1^H-NMR of *L. hyperborea* and partially oxidized alginate

The ^1^H NMR-spectra of partially oxidized alginate and *L. hyperborea* stipe alginate are shown in figure S1. Peaks are assigned according to literature^1^.


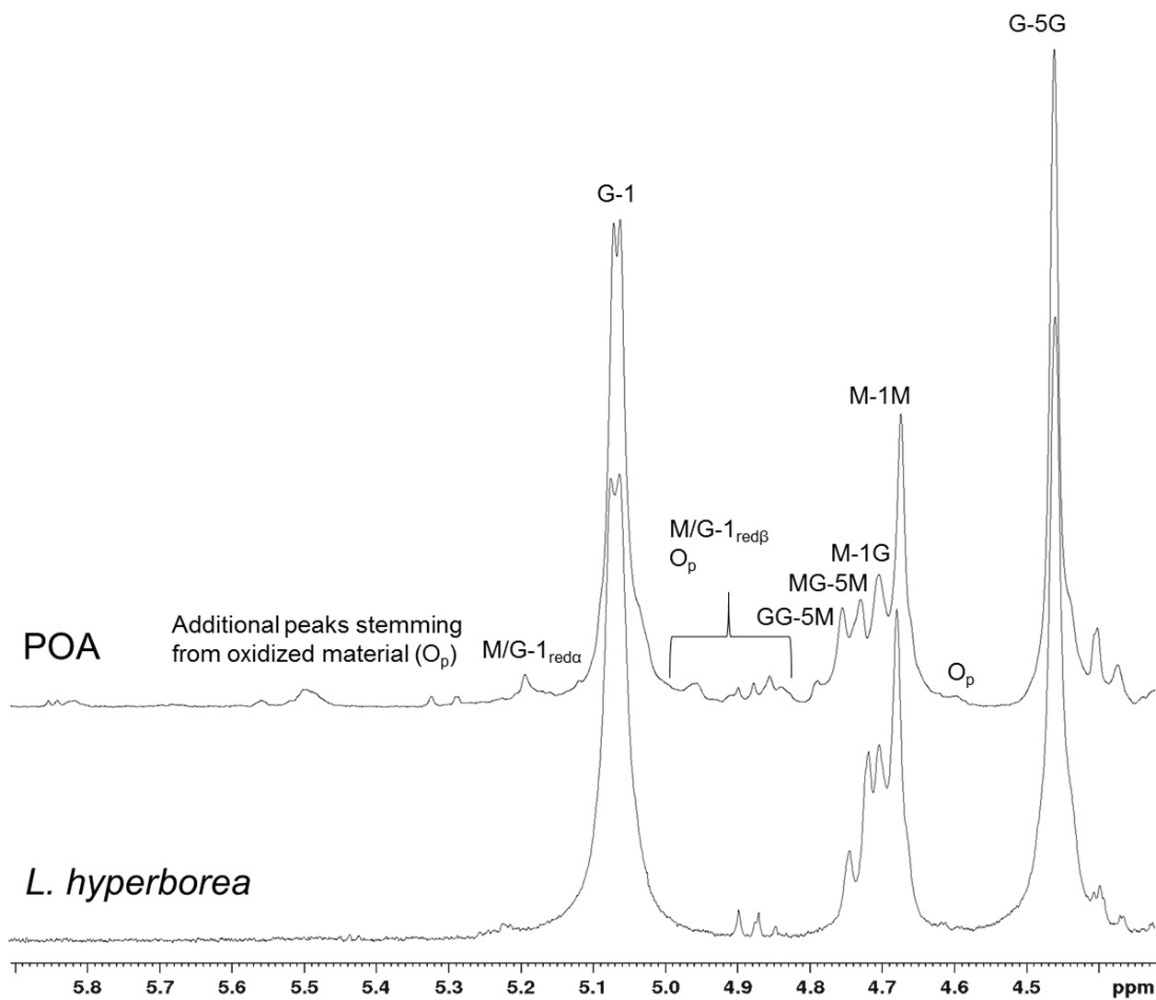


Supplementary figure S1. ^1^H NMR-spectra of partially oxidized alginate (POA, 400 MHz, 90°C) and *L. hyperborea* (300 MHz, 90°C)*.* Peaks denoted with an “O” refers only to the oxidized material.

(1) Kristiansen, K. A.; Schirmer, B. C.; Aachmann, F. L.; Skjåk-Bræk, G.; Draget, K. I.; Christensen, B. E. *Carbohydr. Polym.* **2009**, *77* (4), 725–735.

Supplementary Information B: SEC-MALS data

The concentration profiles and slice molecular weights are shown in figure S2 for one sample of unmodified alginate, partially oxidized alginate, partially oxidized alginate coupled with linker, and β-CyD-grafted alginate.
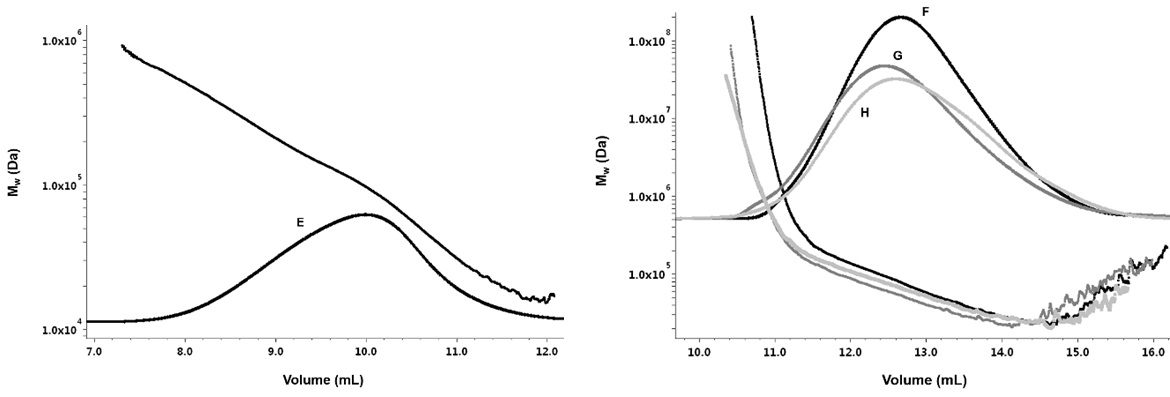


**Supplementary figure S2:** SEC-MALS data. Concentration profiles (solid lines) and slice molecular weights (dotted lines). Data at peak ends were removed due to noise or large deviations possibly due to aggregates prior to analysis. E: unmodified alginate. F: partially oxidized alginate. G: POA coupled with 4-pentyn-1-amine. H: β-CyD-grafted alginate.
